# Embryonic chirality and the evolution of spiralian left–right asymmetries

**DOI:** 10.1098/rstb.2015.0411

**Published:** 2016-12-19

**Authors:** José M. Martín-Durán, Bruno C. Vellutini, Andreas Hejnol

**Affiliations:** Sars International Centre for Marine Molecular Biology, University of Bergen, Thormøhlensgate 55, Bergen, 5006 Norway

**Keywords:** Spiralia, Nodal, Pitx, left–right axis, evolution

## Abstract

The group Spiralia includes species with one of the most significant cases of left–right asymmetries in animals: the coiling of the shell of gastropod molluscs (snails). In this animal group, an early event of embryonic chirality controlled by cytoskeleton dynamics and the subsequent differential activation of the genes *nodal* and *Pitx* determine the left–right axis of snails, and thus the direction of coiling of the shell. Despite progressive advances in our understanding of left–right axis specification in molluscs, little is known about left–right development in other spiralian taxa. Here, we identify and characterize the expression of *nodal* and *Pitx* orthologues in three different spiralian animals—the brachiopod *Novocrania anomala*, the annelid *Owenia fusiformis* and the nemertean *Lineus ruber*—and demonstrate embryonic chirality in the biradial-cleaving spiralian embryo of the bryozoan *Membranipora membranacea*. We show asymmetric expression of *nodal* and *Pitx* in the brachiopod and annelid, respectively, and symmetric expression of *Pitx* in the nemertean. Our findings indicate that early embryonic chirality is widespread and independent of the cleavage programme in the Spiralia. Additionally, our study illuminates the evolution of *nodal* and *Pitx* signalling by demonstrating embryonic asymmetric expression in lineages without obvious adult left–right asymmetries.

This article is part of the themed issue ‘Provocative questions in left–right asymmetry’.

## 1. Introduction

Bilaterally symmetrical animals exhibit two orthogonal main body axes, namely the anteroposterior and the dorsoventral axes, which establish a plane of symmetry that runs longitudinally along the midline of the animal, and defines the left–right axis of the organism [1]. In many species, the left and right body regions are mirror images of each other, and thus there is an exact correlation between the organs and structures on each side. In other organisms, however, body parts develop asymmetrically along the left–right axis [2,3]. We humans exhibit a common example of this situation, with our heart located on the left side of the body.

**Figure 1.**
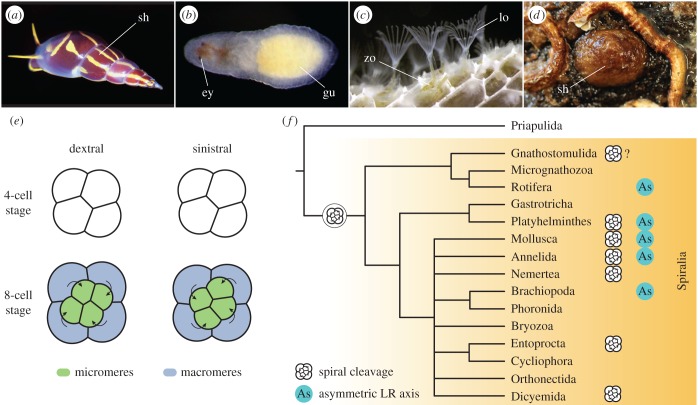
The Spiralia, embryonic chirality and the distribution of cleavage modes. (*a*) The marine snail *Annulobalcis aurisflamma* (credit Alvaro E. Migotto). (*b*) Juvenile nemertean of *Lineus ruber*. (*c*) Adult zooids in a bryozoan colony of *Membranipora membranacea*. (*d*) Adult specimen of the brachiopod *Novocrania anomala*. (*e*) Spiral-cleaving embryos display embryonic chirality at the eight-cell stage. The asymmetric division of the four blastomeres at the four-cell stage forms four animal micromeres that can be shifted either dextrally or sinistrally with respect to the vegetal macromeres. In molluscs, there is a direct correspondence between this chirality and the direction of coiling of the shell and internal organs. (*f*) Distribution of spiral cleavage and left–right asymmetries in Spiralia. Phylogeny according to [4]. In (*e*) and (*f*), drawings are not to scale. Abbreviations: ey, eyes; gu, gut; lo, lophophore; sh, shell; zo, zooid.

One of the most beautiful examples of left–right asymmetries occurs in the direction of coiling of the shell of snails (figure 1*a*). Snails are molluscs and members of the Spiralia, which is one of the two major clades of the Protostomia [4–7]. The Spiralia comprises a broad diversity of animal forms [8,9], including meiofaunal taxa (e.g. rotifers and gastrotrichs) and large macrobenthic organisms (e.g. segmented annelids and ribbon worms; figure 1*b*). There are not only colonial forms, such as bryozoans (figure 1*c*), but also sessile animals, like brachiopods (figure 1*d*), and behaviourally complex animals like octopuses. Moreover, there is also variation in the life cycles, with taxa showing direct development, groups with intermediate larval forms and parasites. This vast developmental, morphological and ecological diversity contrasts with a seeming simplicity of the left–right axis in most spiralian taxa, which is most often symmetrical (table 1). The most extreme asymmetry is that of the shell and internal organs of gastropod molluscs, and to a less extent the digestive system of other molluscs, annelids, brachiopods and rotifers (table 1).

**Table 1.** Left–right asymmetries in adult and larval forms of Spiralia.

group	left–right axis
Gnathostomulida	Symmetrical
Micrognathozoa	Symmetrical
Rotifera	Symmetrical. Asymmetries in the jaws (trophi) in some species, and unpaired gonad often displaced to one side in Monogononta [10]
Gastrotricha	Symmetrical
Platyhelminthes	Symmetrical. Asymmetries in the ciliary band of some polyclad larvae [11], gonads of rhabdocoels [12], and neural morphology/physiology in polyclad larvae and triclads [13,14]
Mollusca	Asymmetry in shell coiling and internal body organization in Gastropoda [15]. Minor asymmetries, mostly affecting the digestive system, in Polyplacophora, Bivalvia and Scaphopoda [12,16].
Annelida	Symmetrical. Asymmetries in buccal apparatus of some polychaetes [2,17] and digestive system of *Capitella teleta* [18]
Nemertea	Symmetrical. Asymmetric eye in paleonemertean larva [19]
Phoronida	Symmetrical
Brachiopoda	Symmetrical. Anus in the right side in the Lingulacea & Discinacea [20]
Bryozoa	Symmetrical. Asymmetry in the colony coiling [2]
Entoprocta	Symmetrical
Cycliophora	Symmetrical
Orthonectida	Symmetrical
Dicyemida	Symmetrical

Despite the absence of major left–right morphological asymmetries in most adult and larval forms, an inferred ancestral feature present in many lineages of the Spiralia is the quartet spiral cleavage, a programme of highly stereotypical cell divisions that displays embryonic chirality (figure 1*e*) [21–23]. With the third round of zygotic divisions, a typical spiral-cleaving embryo becomes eight cells. These divisions are asymmetric and occur in the direction of the animal–vegetal axis, so that four smaller cells (micromeres) and four larger cells (macromeres) form in the animal and vegetal pole, respectively. However, the micromeres do not align completely parallel to the animal–vegetal axis, but shift either dextrally (i.e. to the right) or sinistrally (i.e. to the left) with respect to the macromeres (figure 1*e*). If this first asymmetric division were dextral, the next division would be sinistral and vice versa. The alternation of the left–right orientation of the mitotic spindles during cleavage is what eventually causes a spiral arrangement of the micromeres when observed from the animal pole, hence the name of this mode of cleavage. The dextral chirality is more common and genetically dominant, often the only conformation of a spiralian embryo and thought to be ancestral [22,24,25]. However, some spiral-cleaving species can produce embryos of either chirality [26]. In gastropods, the chirality of the embryo is intimately connected with the left–right asymmetries of the adult, in a way that dextral embryos develop into dextral coiling molluscs and sinistral embryos form sinistral coiling specimens [27,28]. The mechanical manipulation of the embryonic chirality at the eight-cell stage is furthermore sufficient to cause a shift in the final coiling of the animal [29], suggesting that the left–right development in molluscs, and likely other spiralians, is strongly influenced by the earliest cytoskeletal dynamics [27,30].

The advent of molecular studies in gastropod molluscs, however, revealed an additional unexpected role for the Nodal signalling pathway in the development of the left–right axis in spiralian embryos [31]. The TGF-β ligand *nodal* was thought to be an innovation of the Deuterostomia (i.e. sea urchins, hemichordates, and chordates), where key components of this pathway (*nodal*, *lefty* and *Pitx* paralogues) are asymmetrically expressed along the left–right axis and control the proper development of this axis [32–39]. However, the molluscs *Lottia gigantea* and *Biomphalaria glabrata* also have a *nodal* and a *Pitx* orthologue asymmetrically expressed along the left–right axis [31,40]. Furthermore, chemical disruption of the Nodal signalling results in molluscs with uncoiled shells, demonstrating that this pathway also affects the correct development of the left–right axis in these animals. Therefore, the Nodal signalling pathway was present in the ancestor to all bilaterally symmetrical animals and presumably had an ancestral function in the development of left–right morphological asymmetries [31,41]. Since this discovery, orthologues of *nodal* and *Pitx*, but not *lefty*, have been identified in many other spiralian taxa [42–44], and asymmetric expression of these genes has been reported also in the brachiopod *Terebratalia transversa* [42]. Despite this recent progress, the expression of *nodal* and *Pitx*, and its connection with the early embryonic chirality and final left–right morphology is still unknown in most spiralian taxa. Even more importantly, virtually nothing is known about the early embryonic chirality and development of the left–right axis in those spiralian lineages that have lost spiral cleavage (figure 1*f*).

In this study, we characterize the expression of members of the Nodal signalling pathway in three spiralian taxa with different embryogenesis, life histories and adult morphologies, and analyse the embryonic chirality of a biradial-cleaving spiralian. We show asymmetric expression of *nodal* in the brachiopod *Novocrania anomala* (O. F. Müller, 1776), and of *Pitx* in the annelid *Owenia fusiformis* Delle Chiaje, 1844, as well as symmetrical expression of *Pitx* in the nemertean *Lineus ruber* (Müller, 1774). We further describe symmetric expression of *Pitx* in *Priapulus caudatus* Lamarck, 1816, a member of the Priapulida, which seems to be the most evolutionarily conservative taxon in the Ecdysozoa [45,46], the sister group of the Spiralia. Additionally, we provide evidence for embryonic chirality in the bryozoan *Membranipora membranacea* (Linnaeus, 1767), a spiralian that lost the stereotypical spiral cleavage, and thus does not show the early, dextral or sinistral asymmetric cell divisions. Altogether, our findings improve our understanding of the evolution of the Nodal signalling pathway in metazoans and provide a more comprehensive view of the establishment of left–right chirality during spiralian development.

## 2. Material and methods

### (a) Animal collections and embryo fixation

Adult specimens of *N. anomala* were collected from the coasts near Espeland Marine Biological Station (Norway) during the months of September and October. They were spawned as described elsewhere [47]. Gravid specimens of *O. fusiformis* were collected near Station Biologique de Roscoff, and spawned as previously reported [48]. Adult worms of *L. ruber* were collected, maintained and spawned as previously described [49]. Gravid adults of *P. caudatus* were collected from Gullmarsfjorden (Fiskebäckskil, Sweden) during November, and spawned as described elsewhere [46]. Finally, kelp blades with ripe colonies of the bryozoan *M. membranacea* were collected from floating docks in Hjellestadosen (Bergen, Norway), kept in water tanks with constant running seawater and spawned as previously described [50].

For all the different species, embryos at the desired developmental stage were fixed in 4% paraformaldehyde diluted in seawater for 1 h at room temperature. For *P. caudatus*, the eggshell was permeabilized with 0.05% thioglycolate and 0.01% pronase for 30 min at 9°C before fixation. Larval and juvenile stages of *N. anomala* and *L. ruber* were relaxed in 7.4% magnesium chloride before adding the paraformaldehyde. After fixation, samples were washed several times in phosphate buffer saline supplemented with 0.1% Tween 20. Samples were dehydrated through a graded methanol series and stored in pure methanol at −20°C.

### (b) Gene expression analyses

Full-length sequences of *nodal* in *N. anomala*, and *Pitx* in *O. fusiformis*, *L. ruber* and *P. caudatus* were identified from RNAseq data of mixed embryonic stages. Protein alignments were constructed with MAFFT v. 7 [51] and poorly aligned regions were removed with Gblocks v. 0.91b [52]. RAxML v. 8 [53] was used to infer gene orthologies (electronic supplementary material, figure S1). Resulting trees were formatted with FigTree and Illustrator CS6 (Adobe). Fixed embryos of *N. anomala*, *O. fusiformis*, *L. ruber* and *P. caudatus* were used to perform colorimetric whole mount *in situ* hybridization following previously described protocols [46,49]. After developing the signal, samples were stored in 70% glycerol and imaged with an Axiocam HRc connected to an Axioscope Ax10 (Zeiss), using bright field Nomarski optics. Images were analysed with Photoshop CS6 (Adobe), and figure plates made with Illustrator CS6 (Adobe). Contrast and brightness were adjusted always to the whole image and not to specific parts of it.

### (c) Live microscopy of bryozoan development

We transferred cleaving *M. membranacea* embryos to a glass slide coated with poly-l-lysine, where they were mounted under a coverslip sealed with Vaseline. We imaged the slide under a four-dimensional microscope [54] and acquired 60 optical planes of the embryo every 40 s using differential interference contrast.

## 3. Results

### (a) Expression of *nodal* in the brachiopod *Novocrania anomala*

**Figure 2.**
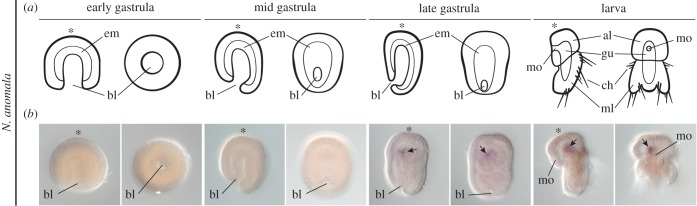
Expression of *nodal* during embryogenesis in *N. anomala*. (*a*) Schematic summary of the embryonic development of *N. anomala* (see §3a for details). (*b*) Whole mount *in situ* hybridization of *nodal* in *N. anomala*. During embryogenesis, *nodal* is first detected in the late gastrula, asymmetrically on the anterior right mesoderm (black arrow). This expression is maintained in the larva (black arrow). For each stage in (*a*) and (*b*), the left drawing/picture is a lateral view and the right drawing/picture is a vegetal/ventral view. Lateral views are oriented with anterior to the left and dorsal to the top. Ventral views are oriented with anterior to the top. The asterisks mark the animal/anterior pole. Drawings are not to scale. Abbreviations: al, apical lobe; bl, blastopore; ch, chaetae; em, endomesoderm; gu, gut; ml, mantle lobe; mo, mouth.

The brachiopod *N. anomala* shows radial cleavage, gastrulation by invagination and the formation of a radially symmetrical gastrula (figure 2*a*) [20,47]. During anteroposterior elongation in the mid and late gastrula, the vegetal blastopore moves posteriorly along the ventral midline and closes (figure 2*a*). After this, the embryo differentiates into a bilobed larva, with an anterior apical lobe, and a posterior mantle lobe with three pairs of chaetae (figure 2*a*). During elongation, the mesoderm forms four pairs of pouches distributed along the anteroposterior axis [20,47]. The first anterior pouch will form the mesoderm of the apical lobe, and the other three consecutive pouches will originate each pair of chaetae bundles.

We identified a single orthologue of *nodal* in *N. anomala* (electronic supplementary material, figure S1*a*). We did not find a clear orthologue of *Pitx* in our transcriptomic data, although *Pitx* is present in the related brachiopod species *T. transversa* [42]. Gene expression analysis during the embryonic development showed that *nodal* was only detected at the end of anteroposterior axial elongation, on the anterior right mesodermal pouch of the late gastrula (figure 1*b*). This expression was maintained in the differentiated larva (figure 1*b*).

### (b) Expression of *Pitx* in the annelid *Owenia fusiformis*

**Figure 3.**
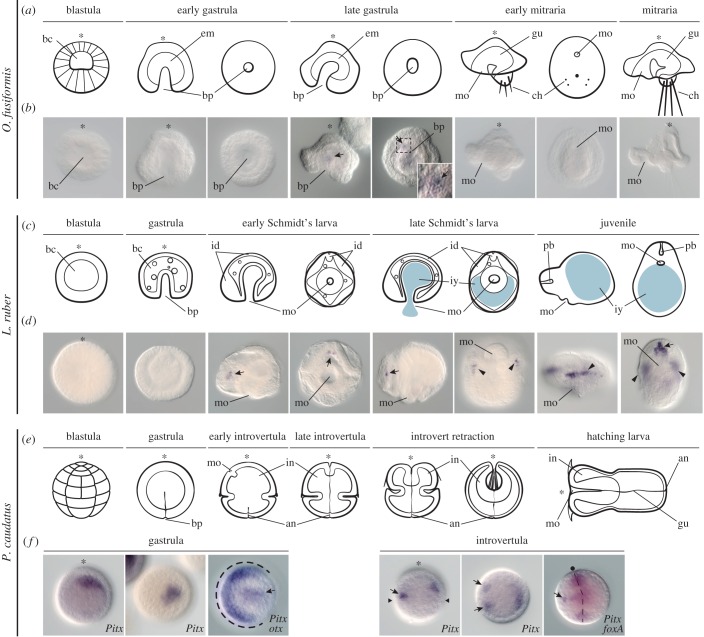
Expression of *Pitx* during embryogenesis in *O. fusiformis*, *L. ruber* and *P. caudatus*. (*a*) Schematic summary of the embryonic development of *O. fusiformis* (see §3b for details). (*b*) Whole mount *in situ* hybridization of *Pitx* in *O. fusiformis*. In the late gastrula, *Pitx* is weakly detected on one mesodermal cell of the right side of the embryo (black arrow). This expression is not retained in the mature mitraria larva. (*c*) Schematic summary of the embryogenesis of *L. ruber* (see §3c for details). (*d*) Whole mount *in situ* hybridization of *Pitx* in *L. ruber*. The first expression of *Pitx* is detected in a pair of symmetrical cell clusters (black arrows) where the proboscis rudiment forms in the Schmidt's larva. In the mature larva, an additional domain in the developing ventral nerve cords appears (black arrohweads). In the juvenile, *Pitx* is expressed in the proboscis (black arrow) and ventral nerve cords (black arrowheads). (*e*) Schematic summary of *P. caudatus* development (see §3d for details). (*f*) Whole mount *in situ* hybridization of *Pitx* in *P. caudatus*. Expression is first observed in endomesodermal cells in the animal pole. Expression of *otx* (dashed line) marks the ventral side. In the introvertula, *Pitx* is expressed in two ventral bilaterally symmetrical cells (arrows) and a cluster of anterior dorsal mesodermal cells. Expression of *foxA* (dashed line) marks the gut and anteroventral mouth (black dot). In (*a*) and (*b*), the blastula stage and the mature mitraria are lateral views, and the other stages are lateral (left) and ventral (right) views. In (*c*) and (*d*), the blastula and gastrula stage are lateral views, and the other stages are lateral (left) and ventral (right) views. In (*e*), all drawings are lateral views. In (*f*), lateral views for each stage are on the left and anterior views are on the right. In all lateral views, the anterior is to the left and dorsal to the top. In all ventral views, the anterior is to the top. The asterisks mark the animal/anterior pole. Drawings are not to scale. Abbreviations: an, anus; bc, blastocoel; bp, blastopore; ch, chaetae; em, endomesoderm; gu, gut; id, imaginal disks; in, introvert; iy, ingested yolk; mo, mouth; pb, proboscis.

The annelid *O. fusiformis* shows stereotypical asymmetric spiral cleavage, with the D quadrant being only slightly larger than the other quadrants [48]. After cleavage, the embryo forms a hollow blastula, and gastrulates by invagination, forming a radial early gastrula (figure 3*a*). At this stage, the internal endoderm bends and forms a U-shape, and the mesoderm grows into two lateral bands [48]. A subequatorial ciliary band forms, together with a bundle of chaetae in the posterior dorsal area, eventually resulting in the formation of the distinctive mitraria larva of oweniids (figure 3*a*) [48,55].

We did not identify an orthologue of *nodal* in our RNAseq data of *O. fusiformis*, but we detected an orthologue of *Pitx* (electronic supplementary material, figure S1*b*). The analysis of the expression of *Pitx* during the embryonic development of *O. fusiformis* showed weak asymmetrical expression in one cell on the right side of the embryo at the late gastrula–early mitraria stage (figure 3*b*). The internal location of the staining suggests that the *Pitx*-positive cell is part of the growing lateral mesodermal bands, as described for the sister species *Owenia collaris* [48]. This expression was restricted to this stage, and not observed in mature mitraria larvae.

### (c) Expression of *Pitx* in the nemertean *Lineus ruber*

The nemertean *L. ruber* shows a characteristic indirect development that involves the formation of an adelphophagic, intracapsular larva [49,56]. Early cleavage is of the spiral type, and results in the formation of a blastula with a small blastocoel. After invagination of the endomesoderm, the radial gastrula develops into the Schmidt's larva (figure 3*c*) [49,56]. This intracapsular larva consists of a temporary epidermis, and a set of epidermal imaginal discs from which the juvenile will form: a pair of cephalic discs, a pair of trunk discs, one proboscis disc, one pharyngeal disc and a blind gut rudiment. The Schmidt's larva can feed on other siblings contained within the same egg capsule, growing in size. After around 18–20 days of development, the larva metamorphoses into the juvenile, which involves the shedding of the larval epidermis, and the differentiation of the juvenile tissues and organs (figure 3*c*).

As with *O. fusiformis*, we identified an orthologue of *Pitx* in the available transcriptomic data (electronic supplementary material, figure S1*b*), but not of *nodal*. The analysis of its expression revealed that Pitx was first expressed symmetrically in a few internal anterior mesenchymal cells of the Schmidt's larva (figure 3*d*). This position corresponds to the place of formation of the proboscis rudiment [49]. In late larval stages, two additional symmetrical domains of expression appeared, which seem to locate where the ventral pair of nerve cords forms (figure 3*d*). After metamorphosis, *Pitx* was detected in the proboscis and ventral nerve cords (figure 3*d*).

### (d) Expression of *Pitx* in the outgroup taxon *Priapulus caudatus*

The priapulid *P. caudatus* exhibits holoblastic radial cleavage [57]. Gastrulation occurs by invagination, and is followed by the division of the embryo in an anterior introvert region and a posterior trunk region (introvertula stage; figure 3*e*) [46]. After differentiation of the larval tissues, the introvert retracts inside the trunk region, and the embryo eventually hatches by protruding the introvert against the hatching cap of the eggshell (figure 3*e*). The first hatching larva is non-feeding, and subsequent rounds of moulting lead to the formation of the definitive adult tissues [58–60].

As in other studied members of Ecdysozoa, *P. caudatus* lacks a *nodal* orthologue [42]. We could identify, however, a *Pitx* gene (electronic supplementary material, figure S1*b*). We detected the first expression of *Pitx* in the gastrula, on a group of endomesodermal cells of the animal pole (figure 3*f*). With the formation of the introvertula, we observed two distinct expression domains: a pair of bilaterally symmetrical ectodermal cells on the ventral side of the introvert, which probably correspond to neural tissue; and a broader expression on the anterior dorsal mesoderm of the introvert (figure 3*f*).

### (e) Embryonic chirality in a biradial-cleaving bryozoan

**Figure 4.**
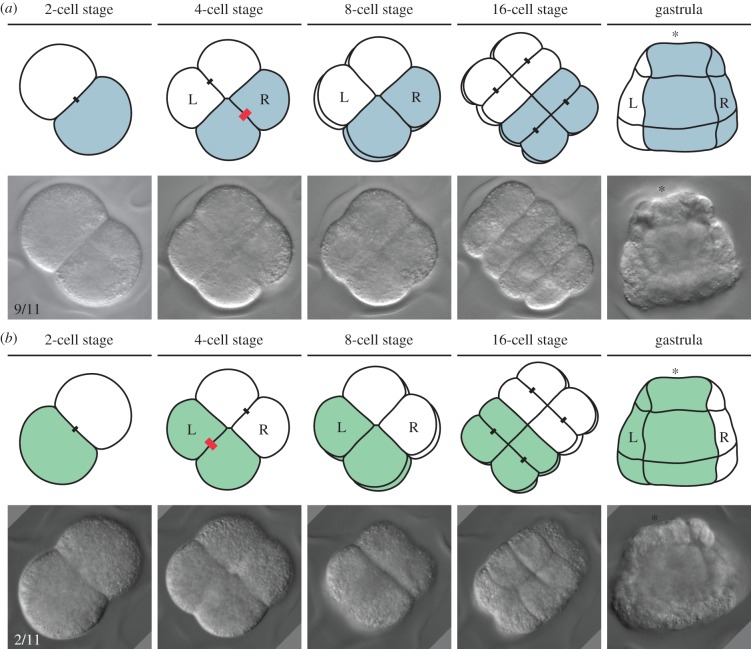
Left–right asymmetries during *M. membranacea* embryogenesis. (*a*) Optical sections of a right-handed embryo in *M. membranacea* (ideally represented in the upper raw; number of observations: 9 of 11 embryos). From the two-cell to the four-cell stages, the cell that will form posterior territories (in colour) cleaves perpendicular to the animal–vegetal axis (thick red bar) and originates the blastomere that will contribute to the right side. (*b*) Optical sections of a left-handed embryo in *M. membranacea* (ideally represented in the upper row; number of observations: 2 of 11 embryos). In a small proportion of embryos, the posterior blastomere at the two-cell stage originates the four-cell stage blastomere from which left tissues develop. Cleaving stages are viewed from the animal pole and the gastrula stage from the posterior region. Drawings are not to scale.

*Membranipora membranacea* shows a stereotypical biradial cleavage pattern where the first and second divisions are meridional, orthogonal to each other and form four equal blastomeres [61,62]. After an equatorial third division, the blastomeres cleave parallel to the plane of the first division forming an eight-by-eight brick-like embryo. Our four-dimensional recordings show that two opposing blastomeres at the four-cell stage give rise to the left and right side of the larval body (figure 4). However, we noticed that in 9 out of 11 embryos, the right blastomere at the four-cell stage is sister to the blastomere giving rise to posterior structures, while in two embryos the pattern is mirrored, the left blastomere is the one sister to the posterior blastomere.

## 4. Discussion

### (a) *nodal*, *Pitx* and the genetic control of left–right development in Spiralia

**Figure 5.**
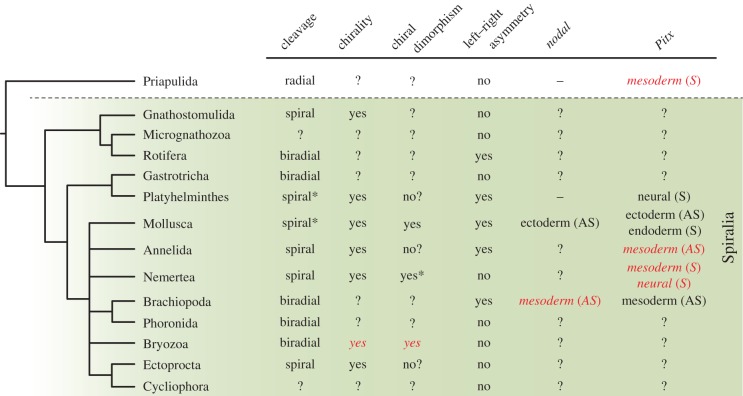
Embryonic chirality and left–right development in Spiralia. Spiral cleavage is probably ancestral to the Spiralia, although this mode of development has been lost in several lineages (the asterisk in Platyhelminthes and Molluscs indicates the presence of non-spiral-cleaving lineages). All spiral-cleaving embryos and some biradial-cleaving embryos (bryozoans) exhibit embryonic chirality. However, this is not always translated into left–right morphological differences in the adult or larval forms. Similarly, the presence of chirality does not always imply intra-specific dimorphism (the asterisk in Nemertea indicates that the dimorphism does not change the final fate of each quadrant). Expression of *nodal* is only known for gastropod molluscs and brachiopods. In both lineages, *nodal* is asymmetrically (AS) expressed, although in different tissues, which does not allow reconstructing the ancestral expression. Expression of *Pitx* is known for flatworms, molluscs, annelids, nemerteans and brachiopods. It can be expressed asymmetrically (AS) or symmetrically (S) and affect different tissues and germ layers. The ancestral expression for the Spiralia is also unclear. Italics, data reported in this study.

The TGF-β ligand *nodal* is asymmetrically expressed along the left–right axis in echinoderms and hemichordates (on the right side), molluscs (on the right or left side, depending on body handedness) and chordates (on the left side), and is functionally required to properly develop this axis in most of these organisms [31–37,39]. Recently, a study showed expression of *nodal* on the right side of the anterior mesoderm in the late gastrula embryo of the rhynchonelliform brachiopod *T. transversa* [42], but its function and influence on the left–right patterning is unknown. In this study, we identified a new *nodal* orthologue in the craniid brachiopod *N. anomala*, but failed to recover a *nodal* member in the annelid *O. fusiformis* and *L. ruber*. However, the presence of *nodal* in other members of the Annelida and Nemertea [42] indicate that these absences are probably not real gene losses, but subsampling transcriptomic issues. The expression of *nodal* in the brachiopod *N. anomala* demonstrated a similar timing and location to that in *T. transversa* (figure 2*b*), albeit these two species differ significantly in the mode of gastrulation and mesoderm development [47,63]. Since the last common ancestor of *T. transversa* and *N. anomala* corresponds to the last common ancestor to all brachiopods [64], our findings indicate that the most probable ancestral expression of *nodal* in brachiopods was in the anterior right, mature mesoderm. This contrasts with the expression in gastropod molluscs, where *nodal* is already expressed at relatively early stages (32-cells) and in ectodermal derivatives of the shell and head region [31]. However, there are no data available on the expression of *nodal* in other groups of molluscs, and in particular, in those without strong left–right asymmetries like the early branching polyplacophorans. Thus, the ancestral expression of *nodal* for this group, and Spiralia generally, is still unclear (figure 5).

The homeobox transcription factor *Pitx* is a downstream regulator of the Nodal signalling pathway, and thus appears asymmetrically expressed on the side of *nodal* expression in members of the Deuterostomia and gastropod molluscs [31,32,36,38]. In the studied molluscs, *Pitx* is additionally expressed in endodermal and cephalic ectodermal domains [31]. In brachiopods, however, *Pitx* is expressed symmetrically, although stronger on the right, *nodal*-positive side of the anterior mesoderm [42]. In platyhelminth species that lack a *nodal* orthologue, *Pitx* is expressed in different neuronal populations, and controls the regeneration of the serotoninergic nervous system and the body midline [42–44]. Our results provide first evidence of expression of *Pitx* in annelids and nemerteans (figure 3*b*,*d*). Interestingly, *Pitx* is expressed symmetrically in the nemertean *L. ruber*, in the nervous system and proboscis, while it is expressed asymmetrically in one anterior right mesodermal cell in the annelid *O. fusiformis*. No expression during early cleavage and development was observed in either of these two spiralians. Altogether, these findings give a complex picture of the evolution of *Pitx* expression in Spiralia (figure 5). When outgroup lineages, such as priapulids (figure 3*f*) are considered, it appears that expression of *Pitx* associated with the nervous system at mid–late stages of development is probably ancestral. However, further analysis of *Pitx* in relation to *nodal* expression in those lineages with both genes will be essential to better understand the evolution of this genetic cassette in spiralians.

Altogether, the expression and functional data on *nodal* and *Pitx* suggest that they are likely involved in the morphological differentiation of the left–right axis in the Spiralia, with asymmetric expression of one or two genes in at least molluscs, annelids and brachiopods (figures 2 and 3) [31,42]. However, the absence of expression of *nodal* and *Pitx* in the earliest cleavage stages in all studied species, when embryonic chirality is established, indicate that a separate upstream genetic mechanism defines the left–right axis in spiralian embryos [29]. In this regard, a recent report showed that a tandemly duplicated, diaphanous-related formin gene (*Ldia2*) is asymmetrically expressed as early as in two-cell stage embryos and maps to the genomic region associated with the inheritance of body handedness in the pond snail *Lymnaea stagnalis* [30]. Formins are involved in actin, and thus cytoskeletal, dynamics [65]. Interestingly, the chemical disruption of this gene during the earliest zygotic divisions leads to the loss of chiral twist in dextral-cleaving embryos [30]. In wild-type sinistral cleaving embryos of *L. stagnalis*, *Ldia2* shows a truncated version. Therefore, these observations suggest that *Ldia2* controls embryonic chirality and that chiral dimorphism evolved with the appearance of a non-functional *Ldia2* recessive allele in *L. stagnalis* [30]. Nonetheless, other mollusc species with sinistral forms do not show the truncated version in their formin genes, which indicates that the genetic basis of embryonic chirality is probably multifactorial. These recent advances are a first step towards understanding the molecular grounds that connect cytoskeleton dynamics and embryonic chirality in spiralian embryos. Further investigations will uncover how these early symmetry breaking events influence the later left–right axis differentiation programme controlled by *nodal* and *Pitx*.

### (b) Embryonic chirality and left–right asymmetries in Spiralia

The dextral or sinistral shift of the animal micromeres, and thus the presence of embryonic chirality, is a defining feature of spiralian cleavage and Spiralia as a whole. However, there are multiple cases of loss of this developmental programme (figure 5), either in major groups (e.g. gastrotrichs, rotifers, brachiopods and bryozoans) or in particular lineages within otherwise spiral-cleaving groups (e.g. in cephalopod molluscs and neoophoran Platyhelminthes) [21,66,67]. Often, the loss of spiral cleavage is associated with the evolution of a radially symmetrical programme of zygotic divisions, with no obvious cellular and/or morphological asymmetries. The bryozoan *M. membranacea* and the brachiopod *N. anomala* display, for instance, this type of development [47,62]. Remarkably, our four-dimensional microscopy approach to study the earliest embryogenesis of *M. mebranacea* demonstrates that there is in fact chiral dimorphism in these biradially cleaving embryos, with the right-handed form being more common than the left-handed, as is also observed in molluscs (figure 4). Whether the same molecular programme involved in controlling embryonic chirality in spiral-cleaving embryos is also playing a role in the early specification of the left–right axis in biradial-cleaving spiralians is unknown.

Altogether, the asymmetric expression of *nodal*/*Pitx* in different lineages, the presence of chiral dimorphism in radial cleaving embryos, and the spiral cleavage itself demonstrate that the presence of left–right asymmetries during development is widespread in the Spiralia. It remains paradoxical, however, that these evident embryonic differences in the cellular fate and molecular profile of the left and right sides are later on not translated into morphological asymmetries in most of the adult and larval forms of the Spiralia.

## 5. Conclusion

Early cytoskeleton dynamics and the subsequent asymmetric activation of the Nodal signalling pathway control the direction of coiling of the shell of gastropod molluscs [29–31], which is one of the most striking cases of left–right asymmetries in animals. Importantly, the presence of embryonic chirality during the first zygotic divisions, which is a defining feature of spiralian development [21,22], is also observed in lineages that have lost the ancestral spiral cleavage, such as the bryozoan *M. membranacea*. Similarly, other spiralians without obvious morphological asymmetries in their adult and larval forms, such as the brachiopods *T. transversa* [31] and *N. anomala*, and the annelid *O. fusiformis*, show asymmetric expression of *nodal* and/or *Pitx* at some point of their embryonic development. Altogether, these evidences indicate that embryonic left–right asymmetries are widespread in the Spiralia, albeit their exact impact on the development of the definitive adult morphology is still unclear.

## Supplementary Material

Figure S1. Gene orthology analyses of nodal and pitx

## Data accessibility

All DNA sequences have been deposited in GenBank (accession numbers KU885445–KU885448).
