# Supplementary figures and images for: Embryonic chirality and the evolution of spiralian left–right asymmetries
Source: Philos Trans R Soc Lond B Biol Sci. 2016 Dec 19;371(1710):20150411. doi: 10.1098/rstb.2015.0411 (PMC5104510; doi:10.1098/rstb.2015.0411)

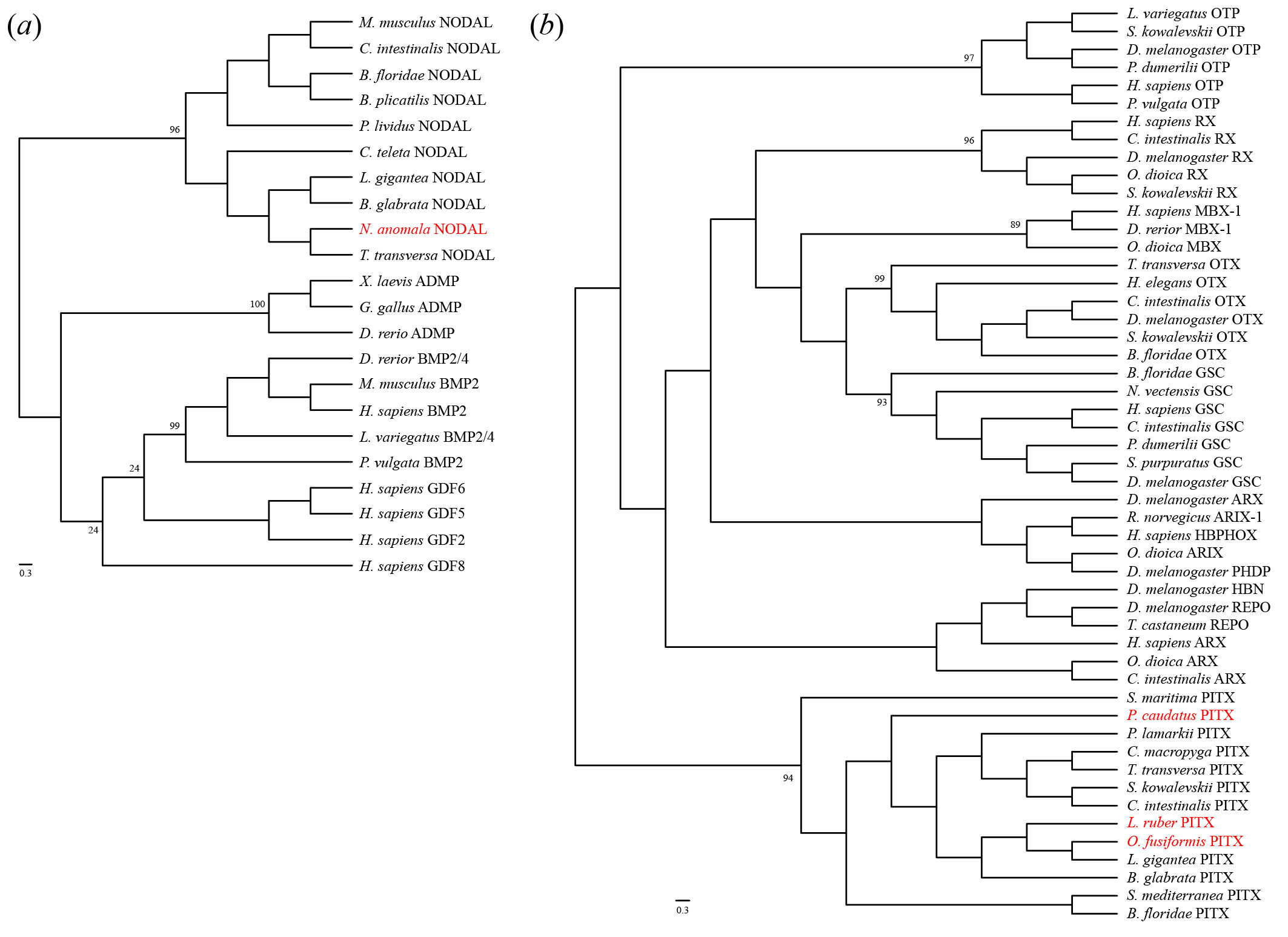

Supplement: Figure S1. Gene orthology analyses of nodal and pitx [file rstb20150411supp1.jpg]
